# Psychiatric Emergency Outreach - an unique opportunity

**DOI:** 10.1192/j.eurpsy.2026.10935

**Published:** 2026-07-31

**Authors:** M. Nordentoft

**Affiliations:** MentalHealth Center, Bispebjerg Hospital, Copenhagen, Denmark

## Abstract

**Introduction:**

Acute psychiatric conditions arising in the community represent a major challenge for health systems, families, and society. These situations can involve severe distress, risk of self-harm or aggression, and disruption of social functioning. They place particular strain on neigbors, relatives and carers. Special initiatives are therefore required to provide timely, person-centered, and effective interventions that can stabilize crises, and reduce harm. Psychiatric Emergency Outreach is an unique initiative in the Capital Region of Denmark.

**Objectives:**

The Danish 10-year plan for psychiatry has allocated state funding to expand acute psychiatric outreach services nationwide. We will present data on how Psychiatric Emergency Outreach serves the target group.

**Methods:**

Since 1997, the Capital Region of Denmark has implemented Acute Psychiatric Outreach, targeting individuals with severe mental illness or those in crisis who cannot resolve their problems through a GP on call or by visiting a psychiatric emergency room. This service is uniquely staffed by a psychiatrist and an ambulance driver, enabling emergency visits even in complex and potentially dangerous situations. Patients, relatives, general practitioners, the police, and social services can directly contact the outreach team, ensuring timely intervention. In cases involving violence or threats, the police collaborate with the team to address the situation safely and effectively. The service addresses challenges faced by severely mentally ill individuals in the community by organizing referrals, facilitating voluntary care, or initiating compulsory admissions when necessary. Data on referrals, response and treatment was registrated in the first two years after initiation and again in 2022-2023. In the first two years of service data of 777 consecutive callers were analysed. In 2022-2023, the sample comprised of 830 callers during daytime.

**Results:**

Most calls to Acute Psychiatric Outreach are highly relevant, involving individuals who require immediate psychiatric intervention. Half of calls solved via telephone consultation. When outreach was organised, one forth of referrals resulted in involuntary admission.

Statistics on referrals, diagnosis, presenting problems, and response will be presented together with clinical cases.

**Conclusions:**

The Capital Region’s model demonstrates how a well-structured acute outreach service can provide timely, expert care to those most in need. As of 2025, this service covers only 40% of Denmark’s population. However, with dedicated funding from the 10-year plan, efforts are underway to expand similar principles and practices to the rest of the country, making acute psychiatric services more accessible nationwide.

**Disclosure of Interest:**

M. Nordentoft Employee of: no conflict

